# Mebendazole-Treated Human Monocyte-Derived Dendritic Cells Represent Promoted Immunogenic Phenotype with Augmented Expression of Inflammatory Markers

**DOI:** 10.34172/apb.025.43394

**Published:** 2025-10-11

**Authors:** Shiva Alipour, Sepideh Sohrabi, Amirhossein Mardi, Negin Karamali, Elham Baghbani, Vahid Khaze, Behzad Baradaran

**Affiliations:** ^1^Immunology Research Center, Tabriz University of Medical Sciences, Tabriz, Iran; ^2^Department of Immunology, Faculty of Medicine, Tabriz University of Medical Sciences, Tabriz, Iran; ^3^Student Research Committee, Tabriz University of Medical Sciences, Tabriz, Iran

**Keywords:** Dendritic cell, Immunotherapy, Mebendazole, Tumor

## Abstract

**Purpose:**

Dendritic cells (DCs) play a critical role in regulating immune responses by influencing the balance between immune tolerance and immunogenicity. While mebendazole (MBZ) is known to polarize macrophages toward a tumor-suppressive phenotype, its effect on DCs remains unclear. This study investigates the effects of MBZ on the phenotype and inflammatory profile of human monocyte-derived dendritic cells (moDCs).

**Methods:**

Peripheral blood mononuclear cells (PBMCs) were separated from the obtained blood from healthy donors using Ficoll density gradient centrifugation. Then, monocytes were isolated via plastic adhesion and subsequently differentiated into moDCs. Cells were treated with MBZ and lipopolysaccharide (LPS) or just LPS, and then surface markers and inflammatory/anti-inflammatory gene expression were measured using flow cytometry and real-time PCR.

**Results:**

The study compared surface marker expression and gene expression of inflammatory and anti-inflammatory cytokines between moDCs and MBZ-moDCs. MBZ-moDCs showed significantly higher CD86 surface expression but lower CD11c and HLA-DR expression in comparison to moDCs. Additionally, MBZ-moDCs exhibited increased interleukin (IL)-12, IL-18, IL-1β, and TNF-α gene levels and decreased IL-10 and IDO levels.

**Conclusion:**

MBZ holds significant potential for reshaping immunotherapy by exerting a profound impact on dendritic cells. By comprehending the intricate interaction between MBZ and dendritic cell function, innovative interventions can be developed.

## Introduction

 Dendritic cells (DCs) are essential constituents of the immune system, strategically dispersed throughout mammalian tissues to orchestrate immune responses. As sentinel immune cells, DCs possess the unique ability to capture, process, and present antigens, thus governing innate and adaptive immune reactions.^1^ This pivotal role renders them crucial for immune surveillance and defense against pathogens, tumors, and other immunological threats. DCs act as specific antigen-presenting cells (APCs) and bridge innate and adaptive immunity.^2,3^ Upon encountering foreign antigens or danger signals, DCs undergo maturation, upregulating co-stimulatory molecules and major histocompatibility complex (MHC) molecules to efficiently present antigens to lymphocytes, particularly T cells, thereby initiating specific immune responses tailored to the encountered antigen.^4,5^ DCs can be categorized into two primary groups: myeloid dendritic cells and plasmacytoid dendritic cells. Myeloid dendritic cells are responsible for stimulating T cells and expressing Toll-like receptors (TLR) 2 and 4, whereas plasmacytoid dendritic cells produce IFN-α and express TLR 7 and 9. Other DCs types include inflammatory DCs, which are derived from precursor monocytes in response to infection or injury. They transform into DCs, characterized by high levels of MHC II expression, various co-stimulatory molecules, and CD11c. Similar to other types of DCs, they can be found in various tissues during inflammatory responses and migrate to nearby lymph nodes. Inflammatory DCs or monocyte-derived DCs (moDCs) play a crucial role in presenting antigens and exhibit similar characteristics to macrophages during inflammation.^4,6,7^ moDCs are commonly used in *in vitro* studies due to their functional similarity to inflammatory DCs present in the tumor microenvironment, accessibility, and ease of generation from peripheral blood monocytes.^8-11^ moDCs can be identified as a distribution of DCs that express certain predominant surface markers, which depict their functional state. CD11c is often used as a broad marker for myeloid DCs and is associated with dendritic lineage and maturation, pointing to the DC phenotype. Also, CD86 is a major co-stimulatory molecule for T cell activation and is indicative of the immunogenic ability of DCs. HLA-DR is a class II MHC molecule and is critical for antigen presentation to CD4⁺ T cells.^12-14^ Each of these markers provides evidence of the activation state and immune-stimulatory ability of moDCs and therefore, were chosen for measurement in this study.

 DCs are sensitive modulators of immune responses, and thus are expected to be a growing target for immunotherapy against cancer and other diseases.^15^ Also, pharmacological agents that can alter DC function for either improved immunogenicity or tolerance have great therapeutic value.^16^ The development of such agents is particularly valuable in clinical settings: drugs that stimulate and mature DCs can strengthen immune responses against tumors or infections, while agents that shift DCs toward a tolerogenic state may help control autoimmune diseases or allergic reactions through enhancing regulatory T cell induction.^17-20^ Mebendazole (MBZ), previously known as an anti-helminthic drug, has recently received attention for its surprising anticancer activities. While the anti-tumor effects of MBZ have been associated with alterations of microtubule dynamics in cancer cells,^21^ current evidence indicates that MBZ may also modify the tumor microenvironment, such as macrophages. It was revealed that MBZ stimulates CD14⁺ myeloid cells, a population that includes monocytes and monocyte-derived cells, leading to enhanced T-cell activation and increased tumor cell killing.^22^ Moreover, there is evidence that MBZ has effects on immune cells, including macrophages,^23^ the potential effects on dendritic cells have been largely unstudied.

 Considering the paramount importance of DCs in connecting innate immunity to a subsequent adaptive immune response, it is important to understand how MBZ affects their phenotype and inflammatory profile. This study aimed to address the existing knowledge gap by evaluating the effects of MBZ on human moDCs, specifically examining changes in surface markers (CD11c, CD86, and HLA-DR), which reflect maturation, co-stimulatory capacity, and antigen presentation, as well as cytokine production, to determine whether MBZ promotes an immunogenic or tolerogenic phenotype. Elucidating these effects may provide novel strategies for cancer immunotherapy and immune modulation.

## Materials and Methods

###  Materials

 In the current study, we prepared a complete medium (CM) containing RPMI 1640, which consists of streptomycin 100 µg/mL, fetal bovine serum (FBS) 15%, penicillin 100 IU/mL, and L-glutamine (2 mmol/L) (all from Gibco, New York, USA). Moreover, recombinant granulocyte-macrophage colony-stimulating factor (rh GM-CSF) and recombinant interleukin-4 (rh IL-4) were purchased from BioLegend (San Diego, United States), as well as 2-mercaptoethanol (2ME) was obtained from Sigma Chemical Co (Munich, Germany). The antibodies employed in the phenotyping of cells were anti-CD86-PE (catalog number: 374205, LOT number: B357748) and anti-HLA-DR-APC (catalog number: 327021, LOT number: B382826) from BioLegend (San Diego, CA, USA), as well as anti-CD14-FITC (LOT number: 114999) and anti-CD11c-FITC (LOT number: 114928) from Immunostep (Salamanca, Spain). Also, Ficoll was achieved from Sigma Chemical Co (Munich, Germany). Lipopolysaccharide (LPS), and MBZ were acquired from Sigma (St. Louis, MO, USA). For RNA extraction, we utilized the TRIzol reagent, which was provided by Roche Diagnostics, Mannheim, Germany. Addscript cDNA synthesis kit (AddBio, Korea) was utilized to synthesize complementary DNA (cDNA), and for evaluation of gene expression by Real-Time PCR, SYBER Green master Mix was provided from Amplicon, England.

###  Isolation of peripheral blood mononuclear cells (PBMCs) and differentiation into DCs 

 Sterile falcons with heparin were utilized to collect peripheral blood from the healthy donor. Ficoll gradient fractionation was used to isolate PBMCs from the 90 cc of blood sample. In 6-well plates, PBMCs were cultured at a density of 5 × 10^6^ cells/mL in serum-free RPMI-1640 medium, and monocytes were separated from PBMCs using the plastic adherence method. After 2 h of incubation at 37°C, non-adherent cells were removed, and adherent cells were cultured in CM with rhIL-4 (25 ng/mL), rhGM-CSF (40 ng/mL), and 2-ME (50 µM). On days 2 and 4, the remaining half of the media was supplied with fresh CM containing rhIL-4 and rhGM-CSF to feed the cultures. 10 μM of MBZ was added on day 5 to the treatment group (2950 µg of MBZ was dissolved in 1000 µL of DMSO, and then 2.5 µL of that solution was added to our 2.5 cc of CM). Following 2 h incubation at 37°C, we added LPS (100 ng/mL) to the culture medium of both of our control and treatment groups to mature DCs generation in 24 hours.

###  Morphology and phenotyping of DCs

 Monocyte and DC morphology were studied and characterized through an inverted light microscope (Optika, XDS-3, Italy). To investigate the phenotype of monocytes and DCs, CD14 (anti-CD14-FITC), CD86 (anti-CD86-PE), HLA-DR (anti-HLA-DR-APC), and CD11c (anti-CD11c-FITC) were used. The cells were stained with the stated antibodies for 30 min in dark conditions and 4 °C. Fluorescence staining was examined using the MACSQuant cytometer (Miltenyi Biotec, CA, USA), and the collected data was processed using FlowJo software v10.5.3.

###  RNA isolation and qRT -PCR 

 The concentration of RNA was evaluated using a spectrophotometer following the extraction of total RNA (A260/280 for RNA of our control group was 1.678 and for RNA of our treatment group was 1.934), which was performed in accordance with the instructions provided by the manufacturer utilizing the TRIzol reagent. Extracted RNA was stored at -80°C, and cDNA was synthesized (the final volume of cDNA synthesis reaction was 20 µL, and the volume of RNA with DEPC water was 11 µL and the volume of reaction buffer was 4 µL, dntp was 2 µL, oligo dT was 1 µL, Random hexamer was 1 µL and Enzyme was 1 µL). The gene expression of inflammatory and anti-inflammatory factors was determined employing the Applied Biosystems StepOnePlusTM Real-Time PCR System (Life Technologies, CA, USA). Then, target mRNAs expression was normalized with the 18s gene as an internal reference. The 2^-ΔΔCt^ technique was utilized to determine relative mRNA expression in all triplicate experiments. The sequences of the primers are presented in Table 1.^24^

**Table 1:** The sequences of the primers of gene ^24^

**Gene**	**Forward/Reverse**	**Sequence**
IDO	F	AGCTTATGACGCCTGTGTGAA
	R	TCCTTTGGCTGCTGGCTTG
IL-12	F	TCAGAATTCGGGCAGTGACTATTG
	R	ATCCTTCTTTCCCCCTCCCTA
TNF-α	F	TTCTCCTTCCTGATCGTGGCA
	R	TAGAGAGAGGTCCCTGGGGAA
IL-18	F	TCTTCATTGACCAAGGAAATCGG
	R	TCCGGGGTGCATTATCTCTAC
IL-10	F	AGGAAGAGAAACCAGGGAGC
	R	GAATCCCTCCGAGACACTGG
IL-1β	F	ATGATGGCTTATTACAGTGGCAA
	R	GTCGGAGATTCGTAGCTGGA
18S	F	ACCCGTTGAACCCCATTCGTGA
	R	GCCTCACTAAACCATCCAATCGG

###  Statistical analysis

 All data was analyzed using GraphPad Prism v9.0.0.121 (GraphPad Software, San Diego, CA, USA). The student’s t-test was used to compare data from the two groups. Each parameter was examined three times for each group, and the results were provided as mean ± SD. A *P* value of ≤ 0.05 was considered significant.

## Results

###  Generation of human monocyte-derived DCs

**Figure 1:**
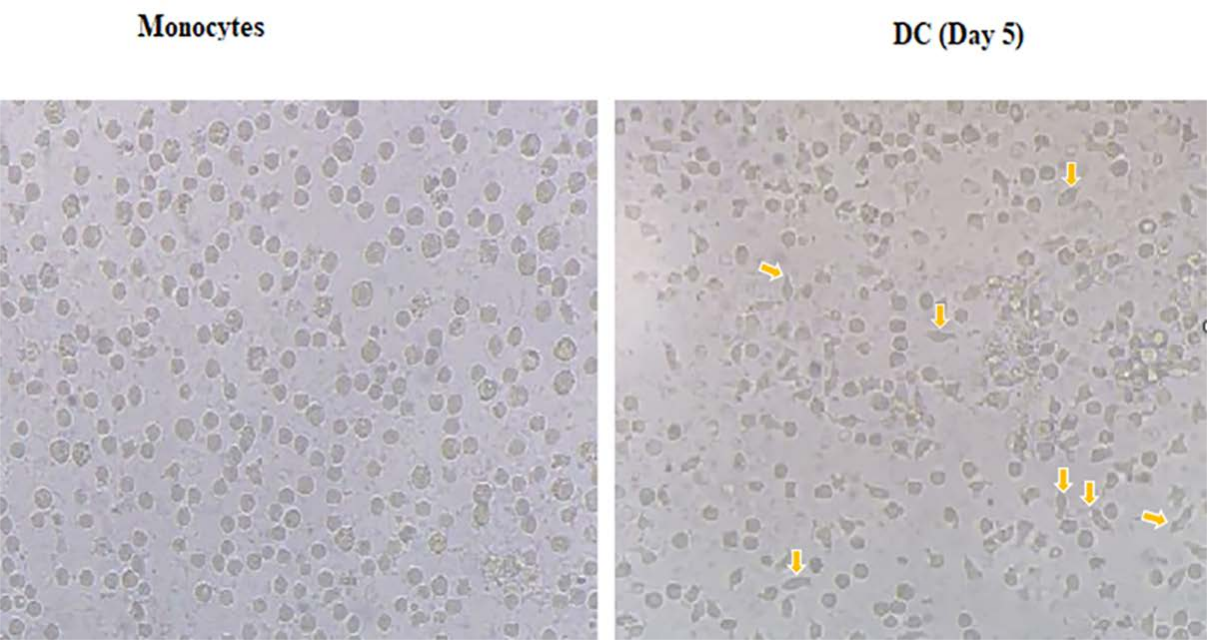
Morphological changes in monocytes and DCs. Monocytes are relatively large round cells and lack appendages, whereas dendritic cells, which function as antigen-presenting cells, possess multiple distinct appendages

 Since monocytes and mature DCs (mDCs) have distinct phenotypical features, morphology changes in these cells were observed by microscopic analysis. Monocytes are characterized by large spherical cells without distinct appendages, while DCs have multiple appendages. As shown, in IL-4 and GM-CSF presence and adding LPS, attached monocyte cells at the plate were differentiated into DCs (Figure 1).

###  Maturation and activation of DCs are affected by MBZ

**Figure 2:**
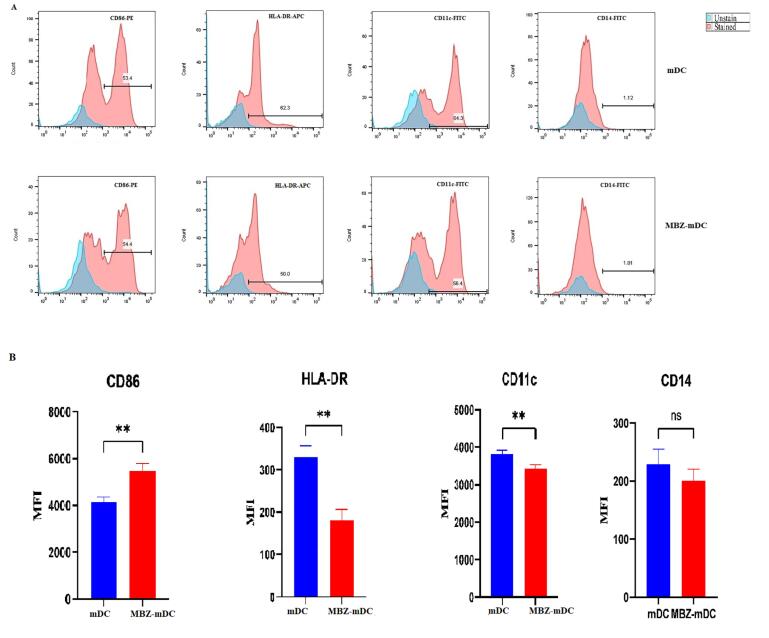
Phenotypical changes between differentiated mDCs and MBZ-mDCs. Flow cytometry was used to determine mDC and MBZ-mDC groups for the expression of surface markers, including CD86, CD11c, and HLA-DR. The results are stated as the percentage of cells expressing these factors **(A)**. Also, surface expression of CD86 (** *P* ≤ 0.01), CD11c (** *P*≤ 0.01), HLA-DR (** *P* ≤ 0.01) and CD14 (ns: non-significant) between MBZ-mDC and mDC are shown as MFI **(B).** MBZ: Mebendazole, mDCs: mature dendritic cells

 We performed phenotypical evaluation of mDCs and MBZ-mDCs groups through using surface factors related to the antigen presentation ability and maturation of DCs, such as CD86, CD11c, HLA-DR, and monocyte marker (CD14). DCs had a low level of CD14 expression, suggesting effective differentiation of monocytes into DCs (Figure 2A). According to the obtained data, the mDCs and MBZ-mDCs groups expressed CD86 (53.4%, 54.4%, respectively), CD11c (64.3%, 56.4%, respectively), and HLA-DR (62.3%, 50.0, respectively) markers (Figure 2A). Then, between two groups of mDCs and MBZ-mDCs, the difference in the surface markers was assessed based on mean fluorescence intensity (MFI). Also, results revealed that surface expression of CD86 (*P* ≤ 0.01**, *P* value = 0.0040, t = 5.960, df = 4) was significantly elevated in the MBZ-mDCs group in comparison to mDCs. While, CD11c (*P* ≤ 0.01**, *P* value = 0.0077, t = 4.959, df = 4), and HLA-DR (*P*≤ 0.01**, *P *value = 0.0023, t = 6.942, df = 4) expression was significantly diminished in the MBZ-mDC group and CD14 expression difference was not statistically significant (Figure 2B).

###  Mebendazole promoted the expression level of inflammatory cytokines and decreased the expression level of anti-inflammatory cytokines 

**Figure 3:**
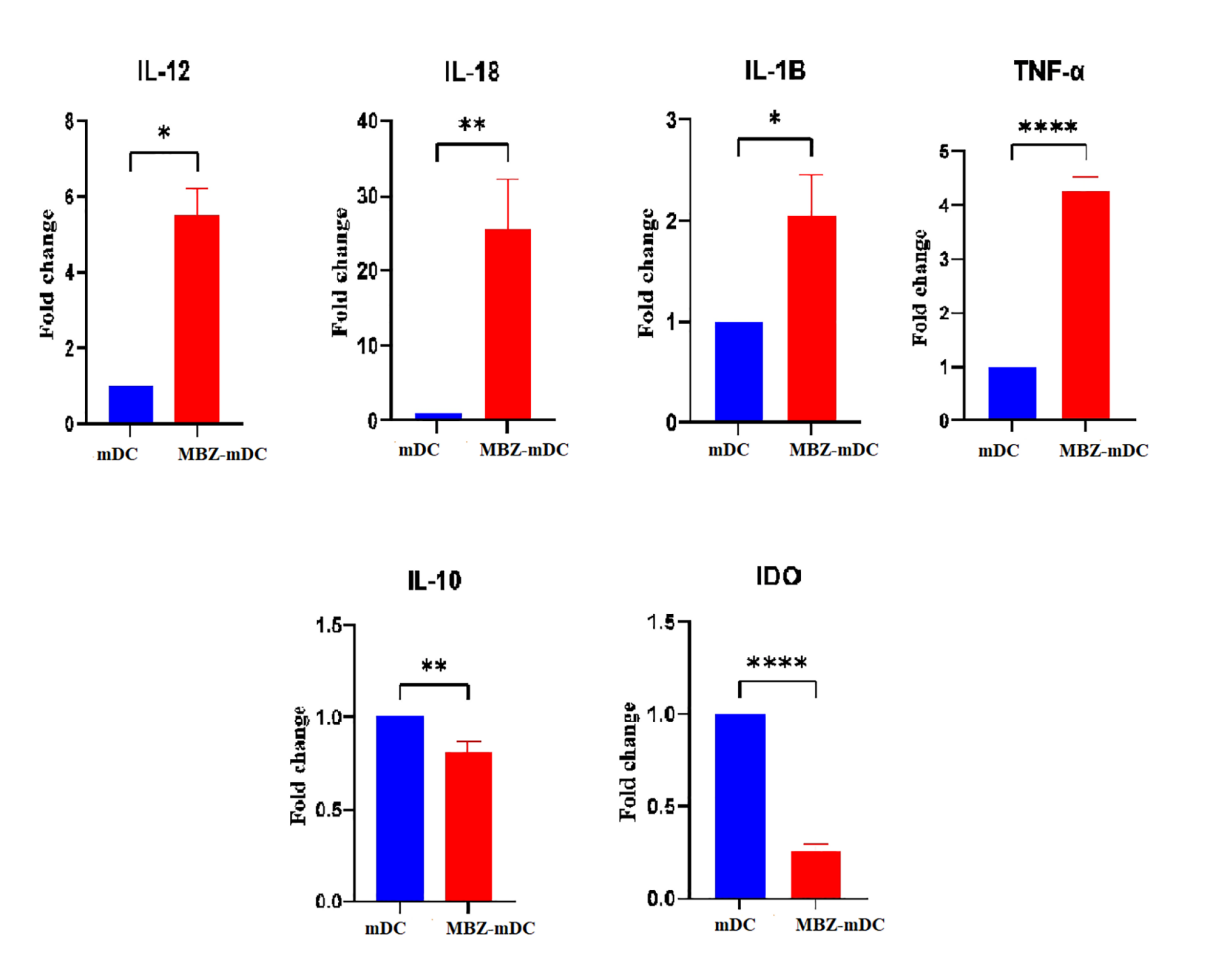
Gene expression profiling of inflammatory and anti-inflammatory cytokines in mDCs and MBZ-mDCs. As shown, expression levels of inflammatory markers, including IL-12 (*P* ≤ 0.05*), IL-18 (*P* ≤ 0.01**), IL-1β (*P* ≤ 0.05*), and TNF-α (*P* ≤ 0.0001****) were remarkably higher in MBZ-mDCs compared to mDCs. Also, anti-inflammatory cytokines’ expression, such as IL-10 (*P* ≤ 0.01**) and IDO (*P* ≤ 0.0001****) in MBZ-mDCs were significantly decreased compared to the mDC group.) DO: Indoleamine 2,3-dioxygenase, IL: interleukin, MBZ: Mebendazole, mDCs: mature dendritic cells, TNF-α: tumor necrosis factor alpha

 Following treatment of DCs with MBZ, the expression level of pro- and anti-inflammatory cytokines was measured by qRT-PCR. According to the results, the expression levels of IL-12 (*P* ≤ 0.05*), IL-18 (*P* ≤ 0.01**), IL-1β (*P* ≤ 0.05*), and tumor necrosis factor alpha (TNF-α) (*P* ≤ 0.0001****) genes were remarkably elevated in MBZ-mDC group in comparison to untreated group (Figure 3). Moreover, the expression levels of IL-10 (P ≤ 0.01**) and IDO (*P* ≤ 0.0001****) was significantly diminished in the MBZ-mDCs group (Figure 3).

## Discussion

 DCs are vital APCs that connect the innate and adaptive immunity, activating T-cells against tumors.^25,26^ They originate from bone marrow precursors called CMPs, giving rise to diverse types with varying localization, surface markers, and functions. In mice, the main DC groups are plasmacytoid dendritic cells, conventional dendritic cells, and myeloid dendritic cells. Inflammation leads to the emergence of moDCs from monocytes. During *Listeria* infection, increased CCL2 prompts the migration of CCR2-expressing monocytes from the bone marrow, resulting in CD11c and MHC II upregulation and moDC differentiation. MoDCs at infection sites produce TNF-α and iNOS, contributing to innate immune defense, and are known as Tip-DCs.^8,27^

 Researchers are currently engaged in the active pursuit of diverse treatment strategies aimed at addressing the constrained functionality of DCs in diseases. These endeavors seek to bolster DC function through a spectrum of methods. Among these, whole-cell DC vaccines constitute a prominent approach, entailing the external maturation and/or expansion of DCs sourced from monocytes or cDC precursors. Notably, moDCs represent a prevailing choice in clinical trials due to their widespread utilization.^28^ In this study, our focus was to examine the impact of MBZ on dendritic cells. Our research findings revealed that the treatment of moDCs with MBZ led to a notable decrease in the expression of the HLA-DR surface marker and an increase in the expression of the CD86 surface marker on the MBZ-moDCs. Elevation in the expression of inflammatory cytokines such as IL-18, IL-12, TNF-α, and IL-1 and a significant reduction in IL-10 levels and IDO are also one of the other findings of this study. The immunomodulatory effects of MBZ on moDCs manifest through altered marker expression and cytokine production. Increased CD86 expression indicates enhanced DC maturation and activation, potentially boosting antigen presentation and T-cell activation. MBZ-moDCs also exhibit increased inflammatory cytokine expression, indicative of pro-inflammatory response, alongside decreased anti-inflammatory cytokine expression, suggesting a shift towards a more pro-inflammatory state. The increase in CD86 expression and decrease in HLA-DR expression after MBZ treatment of moDCs can be explained by the multifaceted effects of MBZ on moDC function. MBZ selectively affects surface markers on moDCs, enhancing CD86 expression involved in T-cell activation while downregulating HLA-DR expression essential for antigen presentation. The effects of MBZ on CD86 and HLA-DR expression depend on the context of moDC activation and the microenvironment. MBZ modulates diverse signaling pathways in moDCs, leading to divergent effects on CD86 and HLA-DR expression. These changes reflect broader shifts in DC phenotype and function, contributing to the immunomodulatory effects of MBZ. Further research is needed to understand the underlying molecular pathways and implications for immune regulation.

 A study by Rubin et alindicated that MBZ induces a tumor-suppressive M1 phenotype in THP-1 monocytes and macrophages. This study further investigates MBZ’s immune effects using human PBMCs co-cultured with tumor cells. MBZ significantly increases TNF-α and IFN-γ, indicating immune stimulation, and enhances pro-inflammatory cytokine release in activated PBMCs. Moreover, MBZ boosts tumor cell apoptosis and reduces surviving tumor cells in PBMC co-cultures, dependent on CD14 monocytes/macrophages.^29-31^ In another study, it was observed that MBZ influences immune activity by activating ERK signaling in monocyte/macrophage

 models. The study found that MBZ increased the activity of ERK in both THP-1 monocytes and PMA-differentiated macrophages. As a result of this activation, downstream effects were observed, including ERK phosphorylation of the substrate P90RSK and the release of IL-1β. These findings suggest that MBZ can modulate immune responses through the activation of the ERK signaling pathway in monocytes and macrophages.^30,32,33^ Yang et al demonstrated that MBZ exerts potent anti-leukemic effects by inducing PANoptosis through activation of Z-DNA-binding protein 1 (ZBP1) signaling in acute myeloid leukemia (AML) cells. This effect was mediated by MBZ targeting the α-tubulin isoform TUBA1A, leading to immune activation and inflammatory cytokine release.^34^ Although this study was performed in AML cells rather than dendritic cells, it underscores MBZ’s ability to modulate intracellular immune-related signaling pathways. In addition, MBZ significantly increases the production of inflammatory cytokines, such as IL-8 and TNF-α, in human monocytic THP-1 cells.^33^

 The contradictory results observed in our study, with the increase in CD86 expression and inflammatory cytokines but a decrease in HLA-DR and CD11c expression, present a challenge in interpreting the findings. Typically, changes in these markers are expected to correlate due to their interconnected roles in DC function. However, discrepancies can arise from various factors influencing DC behavior and marker expression. One potential explanation for these discrepancies is the complexity of DC heterogeneity. moDCs comprise heterogeneous subsets with distinct phenotypic and functional characteristics. Studies have shown that CD16^+^ moDCs express higher levels of surface markers such as CD86, CD11a, and CD11c compared to CD16- moDCs.^35^ Therefore, it is plausible that MBZ treatment selectively impacts specific moDC subsets, resulting in divergent changes in marker expression. Different moDC subsets may possess unique signaling pathways and regulatory mechanisms, leading to varied responses to MBZ.^36^ In addition to aforementioned, another possible explanation is that MBZ may influence DCs phenotype through modulation of intracellular signaling pathways such as STAT3, which has been implicated in DC function and maturation. A recent study in non-small cell lung cancer cells demonstrated that MBZ treatment significantly increases intracellular ROS, which in turn leads to downregulation of phosphorylated STAT3. This ROS-mediated suppression of STAT3 signaling was associated with robust induction of apoptosis and inhibition of cell migration.^37^ Additionally, the dynamic nature of DC maturation and activation is another factor to consider.^38^ Normally, CD86 and HLA-DR expression levels are positively correlated as they increase during DC maturation. However, the observed decrease in HLA-DR expression post MBZ treatment suggests a potential disruption in moDC maturation. This could be influenced by the timing of analysis or other factors affecting DC maturation kinetics and marker expression. Thus, a more detailed analysis involving specific moDC subsets or time-course experiments is required to clarify whether these marker changes occur in different subpopulations or at distinct stages of activation. Communication between DCs and other immune cells, like T cells or macrophages, can impact cytokine production. Changes in the cytokine environment can directly or indirectly affect DCs, potentially leading to conflicting results. Considering the broader immune context and potential crosstalk between different cell types is crucial.^39,40^ To gain a clearer understanding of these contradictory findings, further investigations are warranted. Exploring MBZ’s effects on specific moDC subsets, investigating underlying signaling pathways, and assessing functional consequences such as DC-T cell interactions or antigen presentation assays could offer more insights. Additionally, considering MBZ’s impact on other immune cell populations and the overall immune response would help contextualize observed discrepancies and elucidate broader implications of MBZ treatment on DC function.

 This study has provided new information on the immunomodulatory effects of MBZ on moDCs. Our results showed that MBZ treatment increased expression of CD86 and pro-inflammatory cytokines and decreased HLA-DR and CD11c levels which indicated a complex, and potentially opposite effects on DC maturation. The results of this study suggest that MBZ treatment may alter DC function in a biologically relevant way for immune activation, but it must be cautioned that this study used only *in vitro* human moDCs and did not have any protein or functional assays, such as T cell activation or *in vivo* studies. Thus, MBZ appears to have potential as an immunomodulatory agent; however, further studies using functional studies and *in vivo* models would be needed to assess its potential clinical use in cancer immunotherapy. Moreover, although our protocol aimed to generate a homogeneous population of moDCs, the presence of residual monocytes or apoptotic cells cannot be fully ruled out. Thus, reduction in IL-10 and IDO may partly reflect effects from residual monocytes or apoptotic cells, not solely MBZ’s direct impact on moDCs. Also, our study had limitations due to limited surface marker panel lacking some important maturation and tolerance markers (e.g., CD83, CCR7, PD-L1, and CD40) and our study also failed to assess a relevant signaling pathway (e.g., TLR2, ERK, or NF-κB). These limitations will inhibit mechanistic insight and suggest conclusions about the immunogenic phenotype should be concluded cautiously, not limited to data from CD86 and cytokine expression.

## Conclusion

 Our results suggest that MBZ alters the gene expression profile of human moDCs toward a more immunogenic phenotype. However, since our data was limited to *in vitro* gene expression analysis without supporting protein-level or functional analysis, the potential use of MBZ as a therapeutic agent in immunotherapy is in the early phases. In order to validate these findings and provide insights into the true potential of MBZ as an immunomodulator, further work will need to validate with protein expression and functional assays, including T-cell activation.

## Competing Interests

 The authors declare that there is no conflict of interest.

## Data Availability Statement

 The data that support the findings of this study are available from the corresponding author upon reasonable request.

## Ethical Approval

 The studies involving human participants were reviewed and approved by the ethical code: IR.TBZMED.REC.1401.631.
